# CONNECTING THE DOTS: SUPPORTING OLDER ADULTS LIVING WITH HIV-ASSOCIATED NEUROCOGNITIVE DISORDERS

**DOI:** 10.1093/geroni/igad104.1682

**Published:** 2023-12-21

**Authors:** Elliott Weinstein, Daniel Jimenez, Hetta Gouse

## Abstract

Although people living with HIV are living longer than ever before, significant HIV-related physical and mental health disparities remain among older adults living with HIV (OALWH) due to issues related to advanced aging. HIV-associated neurocognitive disorders (HAND) are becoming increasingly common and have the potential to negatively impact the cognitive, motor, and psychological well-being of OALWH. However, more research on both predictors of HAND and interventions to support cognitively impaired OALWH is needed. This symposium offers insights along the spectrum of intervention development. Two presentations will focus on best identifying predictors of HAND among groups most at-risk up while the other three discuss tailoring and evaluating interventions to alleviate HAND-related symptoms within diverse samples of OALWH from across the country. The first presentation centers on how cerebrospinal fluid markers of Alzheimer’s-related pathology can disentangle diagnosing HAND and amnestic mild cognitive impairment among OALWH in Southern California while the second highlights the role of grip strength in predicting frailty and cognitive impairment among OALWH living in South Florida. The third presentation assesses the feasibility, acceptability, and preliminary intervention effects of a physical activity intervention on cognition adapted for Latino OALWH in South Florida while the fourth examines qualitative feedback from several focus groups on adapting cognitive remediation group therapy as a hybrid group intervention for OALWH with cognitive concerns living in two Canadian provinces. Finally, the last presentation reviews findings of a 2-year randomized clinical trial examining speed of processing training on everyday functioning among OALWH in the U.S. Deep South.

